# Receptor tyrosine kinase-dependent PI3K activation is an escape mechanism to vertical suppression of the EGFR/RAS/MAPK pathway in KRAS-mutated human colorectal cancer cell lines

**DOI:** 10.1186/s13046-019-1035-0

**Published:** 2019-01-28

**Authors:** Pietro Paolo Vitiello, Claudia Cardone, Giulia Martini, Davide Ciardiello, Valentina Belli, Nunzia Matrone, Giusi Barra, Stefania Napolitano, Carmina Della Corte, Mimmo Turano, Maria Furia, Teresa Troiani, Floriana Morgillo, Ferdinando De Vita, Fortunato Ciardiello, Erika Martinelli

**Affiliations:** 1 Department of Precision Medicine, Università degli studi della Campania “Luigi Vanvitelli”, 80131 Naples, Italy; 2 Centro Cellex, Vall D’Hebron Institute of Oncology (VHIO), Barcelona, Spain; 3 MD Anderson Cancer Center, Houston, TX USA; 4 Department of Biology, University of Naples Federico II, 80126 Naples, Italy

**Keywords:** Colorectal cancer, Tumor cell biology, Epidermal growth factor receptor (EGFR), RAS mutation, MAPK pathway, PI3K pathway, Vertical suppression, MEK inhibitor, PI3K inhibitor, AKT inhibitor

## Abstract

**Background:**

Previous studies showed that the combination of an anti-Epidermal growth factor (EGFR) and a MEK-inhibitor is able to prevent the onset of resistance to anti-EGFR monoclonal antibodies in KRAS-wild type colorectal cancer (CRC), while the same combination reverts anti-EGFR primary resistance in KRAS mutated CRC cell lines. However, rapid onset of resistance is a limit to combination therapies in KRAS mutated CRC.

**Methods:**

We generated four different KRAS mutated CRC cell lines resistant to a combination of cetuximab (an anti-EGFR antibody) and refametinib (a selective MEK-inhibitor) after continuous exposure to increasing concentration of the drugs. We characterized these resistant cell lines by evaluating the expression and activation status of a panel of receptor tyrosine kinases (RTKs) and intracellular transducers by immunoblot and qRT-PCR. Oncomine comprehensive assay and microarray analysis were carried out to investigate new acquired mutations or transcriptomic adaptation, respectively, in the resistant cell lines. Immunofluorescence assay was used to show the localization of RTKs in resistant and parental clones.

**Results:**

We found that PI3K-AKT pathway activation acts as an escape mechanism in cell lines with acquired resistance to combined inhibition of EGFR and MEK. AKT pathway activation is coupled to the activation of multiple RTKs such as HER2, HER3 and IGF1R, though its pharmacological inhibition is not sufficient to revert the resistant phenotype. PI3K pathway activation is mediated by autocrine loops and by heterodimerization of multiple receptors.

**Conclusions:**

PI3K activation plays a central role in the acquired resistance to the combination of anti-EGFR and MEK-inhibitor in KRAS mutated colorectal cancer cell lines. PI3K activation is cooperatively achieved through the activation of multiple RTKs such as HER2, HER3 and IGF1R.

## Background

Colorectal cancer (CRC) constitutes a global health problem, ranking as the third most common cancer worldwide [1]. Most deaths from CRC are from metastatic disease, which stands as a not curable disease in most cases. Metastatic CRC (mCRC) characterizes about 25% of patients at the time of diagnosis and about 25–30% of patients during follow-up after locoregional therapy [2]. Although cure is not achievable in the majority of mCRC patients, overall survival improved dramatically in the past three decades thanks to the availability of several chemotherapeutics and targeted agents [3–5]. Between the targeted agents available for mCRC, anti-epidermal growth factor receptor (EGFR) monoclonal antibodies (moAbs) such as cetuximab and panitumumab play a prominent role in the therapeutic strategy by blocking the activation of the EGFR and its downstream intracellular signals, the MAPK and the PI3K pathways [6–8]. However, aberrant activation of downstream signaling pathways, especially those that result in activation of MAPK signaling, such as KRAS, BRAF and NRAS mutations, results in clinical resistance to anti-EGFR-based therapy. These activating mutations can be found either in anti-EGFR agents’ *naive* cancers (primary resistance) or as a consequence to the exposure of the malignancy to such agents (acquired resistance) [9]. In particular, KRAS mutations are present in about 40% of all CRCs at the time of diagnosis and constitute the main mechanism of primary resistance to anti-EGFR agents [10]. For this reason, the detection of KRAS mutations, as well as NRAS and BRAF mutations, predicts a lack of response from anti-EGFR moAbs and is always required before the therapy is started [3]. However, though KRAS mutations are found in a wide range of cancers across nearly all lineages, selective KRAS inhibitors are not yet clinically available [11]. Therefore, research has focused on the inhibition of downstream effectors of KRAS oncoproteins in the MAPK pathway [12]. BRAF inhibitors were shown to be ineffective in treating KRAS-driven CRC because of their lack of activity on KRAS-induced BRAF/CRAF dimers [13]. On the other hand, MEK inhibitors are able to suppress MAPK activation in KRAS-dependent tumours, but this effect is transient as it evokes adaptations in MAPK signaling [14]. In particular, MEK inhibition leads to the relieve of MAPK-dependent negative feedback on the pathway and consequential induction of RTK signaling [15]. This readaptation requires higher concentration of MEK inhibitors to achieve therapeutic response, with great limitation to their clinical use due to poor tolerability, and explains the disappointing results in early clinical studies exploring MEK inhibitors in KRAS-mutated cancers [16]. Moreover, it was demonstrated that PI3K activity is a main predictor of MEK-inhibitor resistance in KRAS-driven colorectal cancer [17, 18] and that the addition of a selective PI3K inhibitor could reverse acquired resistance to MEK-inhibition [19]. Although KRAS is able to directly activate PI3K signaling by binding to p110-PI3K subunit, there is increasing evidence that PI3K activation following MEK inhibition is correlated to RTK activity, paving the way to the use of RTK inhibitors in KRAS mutated CRC [20]. With this respect, two different papers demonstrated that co-targeting of EGFR and MEK overcomes both acquired and primary resistance to anti-EGFR agents in CRC cellular models [21, 22]. The approach of targeting multiple knots on the same signaling pathway, defined *vertical suppression*, was also shown to be able to prevent the onset of resistance to anti-EGFR monoclonal antibodies in CRC by intercepting multiple mechanisms of acquired resistance to such agents [23]. In the present study we have investigated the mechanisms that eventually lead to resistance to the vertical suppression of MAPK pathway through combination of EGFR and MEK inhibition in a cellular model of primary resistance to anti-EGFR therapy constituted by KRAS mutated CRC cell lines.

## Materials and methods

### Drugs and chemicals

The MEK1/2 inhibitor BAY-869766 (Refametinib) was kindly provided by Bayer Pharma Italy; the PI3Kα inhibitor GDC-0941 (Pictilisib) and the AKT-inhibitor GDC-0068 (Ipatasertib) were purchased from Selleckchem. Drugs were dissolved in sterile dimethylsulfoxide (DMSO) at 10 mM stock solution concentration and stored in aliquots at − 20 °C. Cetuximab, an anti-EGFR human-mouse chimeric moAb was kindly provided by Merck Italy, Rome. Working concentrations were diluted in culture medium just before each experiment.

### Cell lines and generation of resistant cell lines

Human HCT116, HCT15, LOVO, SW480 CRC cancer cell lines were obtained from the American Type Culture Collection (ATCC) and authenticated by IRCCS “Azienda Ospedaliera Universitaria San Martino-IST Istituto Nazionale per la Ricerca sul Cancro, Genova,” Italy. Cells were grown in RPMI- 1640 (Lonza), supplemented with 10% FBS and 1% penicillin/streptomycin, in a humidified incubator with 5% of carbon dioxide (CO_2_) and 95% air at 37 °C and were routinely screened for the presence of mycoplasma (Mycoplasma Detection Kit; Roche Diagnostics). Resistant cell lines were generated by exposing cell culture to increasing concentration of cetuximab and BAY-879766 at a constant ratio of 1 μg/ml:1 μmol/l.

### Proliferation assay and combination index

Cell proliferation was analysed by the 3-(4, 5-dimethylthiazol-2-yl)-5-(3-carboxymethxyphenyl)-2-(4sulfophenyl)-2H-tetrazolium (MTT) assay. Cell suspensions (500 μl) containing 2000 viable cells were plated in 48 multi-well plates. After 24 h, cells were treated with different concentrations of drugs either as single agents or in combination for 96 h. The IC_50_ and Combination index (C.I.) values were determined by using the Calcusyn program (Biosoft) and plotted in dose response curves. Results represent the median of the three experiments, each performed in quadruplicate.

### Protein expression analysis

Protein lysates containing equal amount of proteins, measured by a modified Bradford assay (BIORAD, Hercules, CA), were subjected to direct Western Blot (WB). Immuno-complexes were dectected with the enhanced chemiluminescence kit (ECL plus, Thermo Fisher Scientific, Rockford, IL). We used the following antibodies from Cell Signalling (Beverly, MA): anti-EGFR, anti-phospho-EGFR (Tyr1068), anti-HER2, anti-phospho-HER2 (Tyr1248), anti-HER3, anti-phospho-HER3 (Tyr1289), anti-IGF1R-beta, anti-phospho-IGF1R-beta (Tyr1135), anti-p44/42 MAPK, anti-phospho-p44/42MAPK, anti-AKT, anti-phospho-AKT (Ser 473), anti-AXL, anti-c-MET, anti-S6 ribosomal protein, anti-phospho-S6 ribosomal protein, anti-4EBP1, anti-phospho-4EBP1, anti-vimentin, anti-E-cadherin, anti-Snail. Anti-α-tubulin (internal loading control) was from Sigma (Sigma-Aldrich, St. Louis, MO). The following secondary antibodies from Biorad were used: goat anti-rabbit IgG and rabbit anti-mouse IgG. Each experiment was done in triplicate.

### Migration and invasion assay

Transwell chambers (6,5 mm diameter, 8 μm pore size polycarbonate membrane, Corning) were used to evaluate the migratory and invasion capacity of parental and resistant cell lines. For the migration assays, 5 × 104 cells were added into the upper chamber of the insert. For the invasion assays, 1 × 105 cells were added into the upper chamber of the insert pre-coated with Matrigel (BD Bioscience). In both assays, cells were plated in medium without serum, and medium containing 10% FBS in the lower chamber served as chemo-attractant. After incubation for 48 h and 72 h, respectively for the migration and invasion assays, the cells that did not migrate or invade through the pores were carefully wiped out with cotton wool. Then the inserts were stained with crystal violet.

### Oncomine comprehensive assay

Samples from HCT116, HCT15 and LOVO parental and resistant cell lines were analyzed by using a targeted high-multiplex PCR-based NGS panel (OncoMine Comprehensive Assay) coupled with high-throughput sequencing using Ion Proton sequencer. DNA (20 ng) was extracted using QIAamp DNA Mini Kit (Qiagen, Crawley, West Sussex, UK) and RNA (10 ng) was extracted RNA extraction was performed by the RNeasy Kit (Qiagen, Crawley, West Sussex, UK) following manufacturer’s instructions and then processed according to manufacturer’s instruction. The panel screens 143 genes and is able to detect 148 single-nucleotide variants, 49 insertions or deletions, 40 copy number aberrations and a subset of gene fusions. The OncoMine Comprehensive Assay analysis includes: 73 hotspot genes (hotspot coverage): *ABL1, AKT1, ALK, AR, ARAF, BRAF, BTK, CBL, CDK4, HEK2, CSF1R, CTNNB1, DDR2, DNMT3A, EGFR, ERBB2, ERBB3, ERBB4, ESR1, EZH2, FGFR1, FGFR2, FGFR3, FLT3, FOXL2, GATA2, GNA11, GNAQ, GNAS, HNF1A, HRAS, IDH1, IDH2, IFITM1, IFITM3, JAK1, JAK2, JAK3, KDR, KIT, KNSTRN, KRAS, MAGOH, MAP2K1, MAP2K2, MAPK1, MAX, MED12, MET, MLH1, MPL, MTOR, MYD88, NFE2L2, NPM1, NRAS, PAX5, PDGFRA, PIK3CA, PPP2R1A, PTPN11, RAC1, RAF1, RET, RHEB, RHOA, SF3B1, SMO, SPOP, SRC, STAT3, U2AF1, XPO1;* CDS, 26 full genes*: APC, ATM, BAP1, BRCA1, BRCA2, CDH1, CDKN2A, FBXW7, GATA3, MSH2, NF1, NF2, NOTCH1, PIK3R1, PTCH1, PTEN, RB1, SMAD4, SMARCB1, STK11, TET2, TP53, TSC1, TSC2, VHL, WT1;* 49 copy number variations*: ACVRL1, AKT1, APEX1, AR, ATP11B, BCL2L1, BCL9, BIRC2, BIRC3, CCND1, CCNE1, CD274, CD44, CDK4, CDK6, CSNK2A1, DCUN1D1, EGFR, ERBB2, FGFR1, FGFR2, FGFR3, FGFR4, FLT3, GAS6, IGF1R, IL6, KIT, KRAS, MCL1, MDM2, MDM4, MET, MYC, MYCL, MYCN, MYO18A, NKX2–1, NKX2–8, PDCD1LG2, PDGFRA, PIK3CA, PNP, PPARG, RPS6KB1, SOX2, TERT, TIAF1, ZNF217* and 22 fusion drivers*: ALK, RET, ROS1, NTRK1, ABL1, AKT3, AXL, BRAF, CDK4, EGFR, ERBB2, ERG, ETV1, ETV4, ETV5, FGFR1, FGFR2, FGFR3, NTRK3, PDGFRA, PPARG, RAF1*.

### RNA extraction and analysis

Total RNA was extracted using Trizol reagent (Life Technologies). The RNA was quantified and analysed for integrity using Nanodrop (Thermo Scientific, Wilmington, DE). Reverse transcriptase reaction was carried out to convert 1 μg of isolated RNA into cDNA using SensiFast reverse transcriptase (Bioline) according to the manifacturer instruction. Expression levels of genes encoding for EGFR, HER2, HER3 and IGF1R were analyzed using Real time quantitative PCR. Gene-specific primers were designed by using PRIMER EXPRESS software (Applied Biosystems). Amplifications were conducted using the SYBR Green PCR Master Mix (Applied Biosystems). The thermal cycling conditions were composed of 50 °C for 2 min (stage 1) followed by a denaturation step at 95 °C for 10 min (stage 2) and then 40 cycles at 95 °C for 15 s and 60 °C for 1 min (stage 3). All samples were run in duplicate, in 25 μL reactions using a quant studio 7 flex (Applied Biosystems) and relative expression of genes was determined by normalizing to 18S, used as internal control gene; to calculate relative gene expression in value it was used the 2- ΔCt or 2- ΔΔCt method. Nonspecific signals caused by primer dimers were excluded by dissociation curve analysis and use of non-template controls. Exact primer sequences are available upon request.

### Microarray

RNA and DNA extraction were performed as previously described. Agilent microarray analyses were performed to assess baseline gene expression profile for HCT116 and LOVO parental cell lines and their resistant clones, HCT116 CM-R and LOVO CM-R, using a one color labeling microarray system. Data were extracted from slide image using Agilent Feature Extraction software (v.10.5). The raw data and associated sample information were loaded and processed by Gene SpringV R 11.53 (Agilent Technologies, CA). For identification of genes significantly altered in resistant cells, total detected entities were filtered by signal intensity value (upper cut-off 100th and lower cut-off 20th percentile) and flag to remove very low signal entities. Data were analyzed using Student’s t test (*p* < 0.05) with a Benjamani–Hochberg multiple test correction to minimize selection of false positives. Of the significantly differentially expressed RNA, only those with greater than twofold increase or twofold decrease as compared to the controls were used for further analysis. The significance of the association between the data set and the canonical pathway was measured in two ways: (i) Ratio of the number of genes from the dataset that map to the pathway divided by the total number of genes that map to the canonical pathway is displayed; (ii) Fisher’s exact test was used to calculate a *p* value determining the probability that the association between the genes in the dataset and the canonical pathway is explained by chance alone.

### RNA interference

The small inhibitor RNAs (siRNAs) ErbB2/HER2, ErbB3/HER3 and IGF1R were from Thermo-Fisher (Thermo Fisher Scientific, Rockford, IL). The siCONTROL Non-targeting Pool (Dharmacon) was used as a negative (scrambled) control. Cells were transfected with 100 nmol/L siRNAs using Hiperfect reagent (Qiagen) following manufacturer’s instructions. The day before transfection, cells were plated in 35 mm dishes at 40% of confluence in medium supplemented with 5% FBS without antibiotics. Cells were harvested 72 h after transfection. Western blot analysis for target protein expression was performed as described above.

### Indirect immunofluorescence

Parental and resistant cell lines were fixed with 4% paraformaldehyde for 10 min, permeabilized in 0.5% Triton X-100 for 10 min and blocked in phosphate-buffered saline buffer (PBS) supplemented with 3% bovine serum albumin (BSA) for 30 min. After each step, the cells were rinsed in PBS, incubated for 1 h at room temperature with primary antibodies anti-EGF Receptor (Cell signaling), anti-HER2 (Cell signaling) followed by incubation with secondary antibodies Alexa Fluor 488 goat anti-mouse IgG (ThermoFisher) or Alexa Fluor 532 goat anti-rabbit IgG (ThermoFisher) for 30 min at RT. Cell nuclei were stained with 4,6-diamidino-2-phenylindole (DAPI). Samples were examined under the fluorescence confocal microscope Zeiss LSM 700 (Zeiss, Oberkochen, Germany), using a 60x oil immersion objective. Images were acquired with a 1024 × 1024 pixels resolution.

### Statistical analysis

The statistical analyses of in vitro and in vivo data were carried out using Prism version 4.02 (GraphPad Software, Inc). The Student t test was used to evaluate the statistical significance of the results. All *P* values represent two-sided tests of statistical significance with P value < 0.05.

## Results

### Establishment and characterization of human KRAS-mutant CRC cell lines resistant to combined treatment with cetuximab and refametinib

**Fig. 1:**
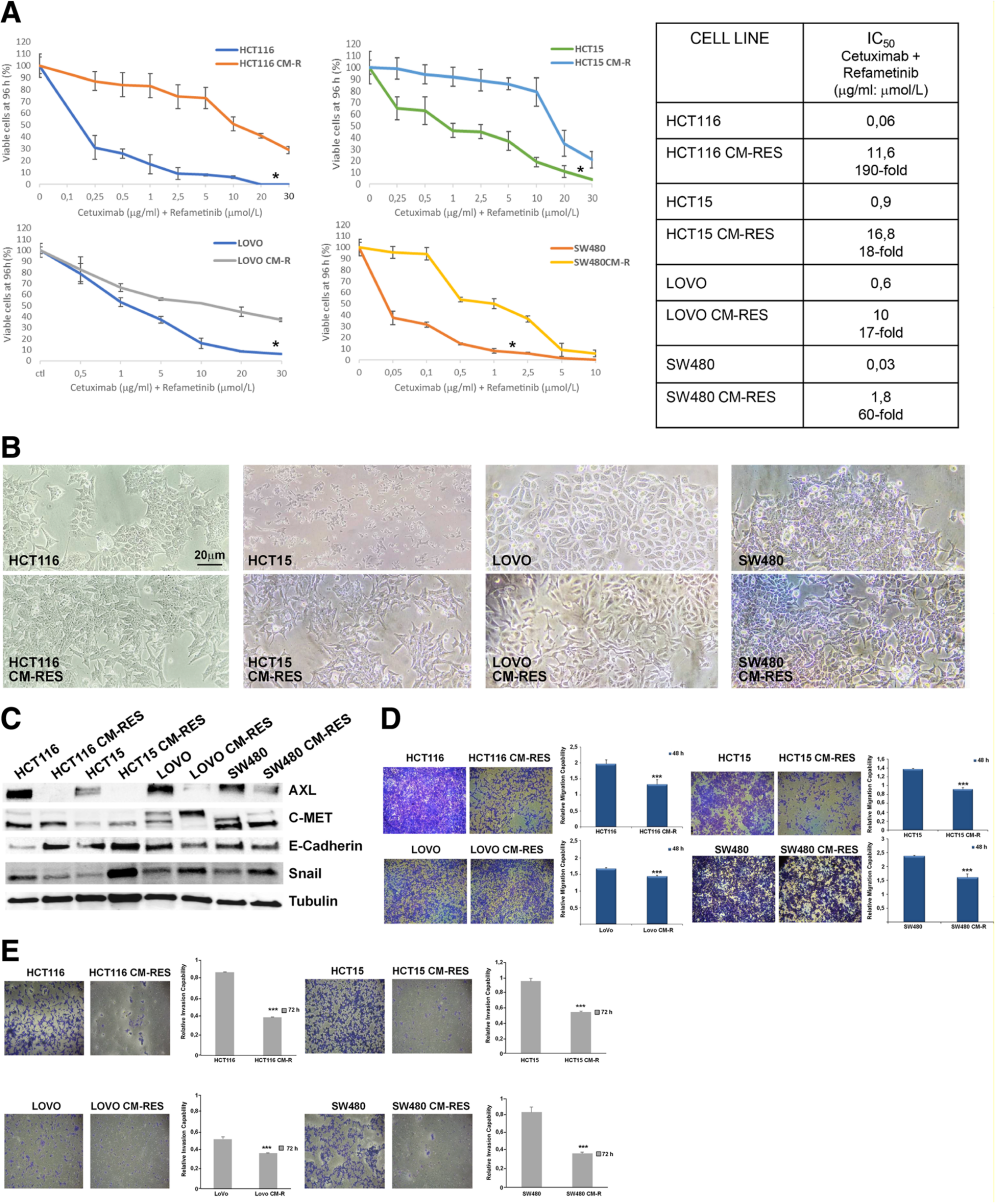
Establishment and characterization of cetuximab-MEKi-resistant (CM-RES) human colorectal cancer cell lines. **a**, sensitivity of parental and resistant cell clones to the combination of cetuximab and the MEK-inhibitor BAY-86-9766 (refametinib) at fixed ratio 1 μg/ml:1 μmol/L after 96-h treatment, evaluated for proliferation by MTT assay, as described in the Materials and Methods. All the results are average ± SD of 3 independent experiments, each done in triplicate. The table summarizes the values of the IC_50_ and the relative fold-change in resistant versus parental cell lines. *: *p* < 0.05. **b**, morphologic changes in resistant cell lines compared to parental cell lines. Magnification 200X, reference bar: 20 μm. **c**, western blot analysis of protein involved in epithelial-mesenchymal transition in baseline condition. **d,** Transwell migration assay at 48 h of parental cells compared to resistant clones. Bars indicate fold-change ± SD compared to parental control. Each assay was performed in triplicate. Magnification: 10X. ***: *p* < 0.001. **e,** Transwell matrigel invasion assay at 72 h of parental cells compared to resistant clones. Bars indicate fold-change ± SD compared to parental control. Each assay was performed in triplicate. Magnification: 10X. ***: *p* < 0.001

We first identified the half-maximal inhibitory concentration (IC_50_) to the combination of the anti-EGFR moAb cetuximab and the selective MEK1/2 kinase inhibitor BAY-879766 (refametinib) used at a fixed ratio in order to achieve a synergistic effect as previously described [22] in a panel of 4 different KRAS-mutated human CRC cell lines: HCT116, HCT15, LOVO, SW480. Notably, two of these cell lines, also carry PIK3CA mutations, respectively H1047R for HCT116 and E545K for HCT15. Cell lines were subsequently cultured under continuous exposure to increasing concentration of the two drugs for 6–9 months, until the emergence of cetuximab-MEK-inhibitor-resistant (CM-R) subclones. IC_50_ for the drug combination is increased in the resistant clones between 17 and 190-fold (Fig. 1a). An evident modification of cellular morphology is associated to the acquired resistance to cetuximab and refametinib (Fig. 1b). Protein expression of different epithelial and mesenchymal markers also changes between parental and resistant clones (Fig. 1c). In particular, AXL levels – a marker related to epithelial-mesenchymal transition –are strongly decreased in the resistant cell lines, while c-MET levels decrease either in the mature (145 kDa, lower band) or in the precursor form (pre-c-MET, 190 kDa, upper band) respectively in LOVO and SW480 resistant cell lines, suggesting different underlying mechanisms. On the other hand, though E-cadherin – a common epithelial marker – is strongly increased both in HCT116 and in HCT15 cell lines, the mesenchymal marker Snail is decreased only in HCT116 resistant cell lines, while it shows a clear increase in HCT15, LOVO and SW480 resistant cell lines. Migration capability at 48 h is homogenously decreased across the 4 resistant clones when compared to parental cell lines (Fig. 1d). Moreover, the invasion ability of the resistant clones is significantly decreased, as evidenced by the Matrigel invasion assay (Fig. 1e). To further characterize the resistant phenotype in the established cell lines, we also performed OncoMine Comprehensive Assay that is able to identify a wide range of common genetic alterations in form of single-nucleotide variants, insertions or deletions, copy number aberrations and a gene fusion. This assay revealed no new genetic events and no significant difference in mutational frequency in the resistant cell lines, compared to parental cell lines (Table 1).

**Table 1:** Oncomine Comprehensive Assay

Name	DNA		RNA
	MUT	CNV	Fusion
HCT116	CTNNB1: p.S45del (c.133_135delTCT) (52,1%); PIK3CA: p.H1047R (c.3140A > G) (45,8%) NOTCH1: p.D1609_R1633del25 (c.4826_4900del75) (33,3%); KRAS: p.G13D (c.38G > A) (53,2%)	**–**	**–**
HCT116 CM-R	CTNNB1: p.S45del (c.133_135delTCT) (41%); PIK3CA: p.H1047R (c.3140A > G) (43%) NOTCH1: p.D1609_R1633del25 (c.4826_4900del75) (32%); KRAS: p.G13D (c.38G > A) (49%)	**–**	**–**
HCT 15	PIK3CA: p.E545K (c.1633G > A) (100%); KRAS: p. p.G13D (c.38G > A) (52,4%); TP53: p.S241F (c.722C > T) (49,2%)	**–**	**–**
HCT15 CM-R	PIK3CA: p.E545K (c.1633G > A) (100%); KRAS: p. p.G13D (c.38G > A) (51%); TP53: p.S241F (c.722C > T) (48%)	**–**	**–**
LoVo	FBXW7: p.R505C (c.1513C > T) (45%); KRAS: p. p.G13D (c.38G > A) (69%);	**–**	**–**
LoVo CM-R	FBXW7: p.R505C (c.1513C > T) (49,6%); KRAS: p. p.G13D (c.38G > A) (78%);	**–**	**–**

The analysis of 148 single nucleotide variants, 49 copy number variations and 22 fusion drivers was negative for new genetic events in resistant vs parental cell lines

### Emergence of AKT pathway activation upon vertical suppression of EGFR and MEK in human KRAS-mutant CRC cell lines

**Fig. 2:**
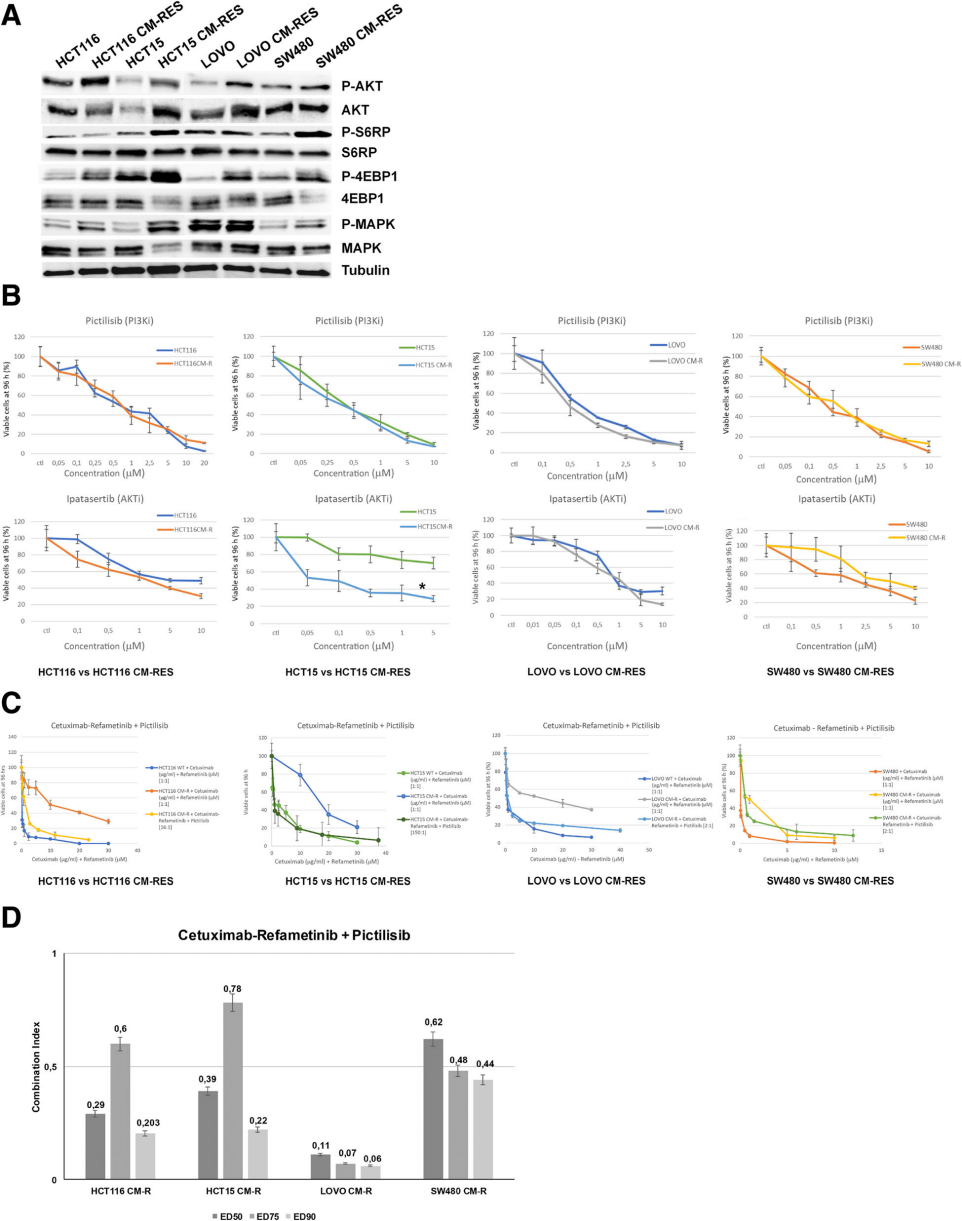
Intracellular pathways and selective inhibition of PI3K/AKT axis. **a**, western blot analysis of intracellular transducers and their phosphorylation status at baseline conditions in parental versus resistant cell lines; AKT and its downstream effectors S6RP and 4EBP1 result activated preferentially in CM-RES clones. Tubulin was used for normalization of protein extract content. **b**, sensitivity of parental and resistant cell clones to treatment with the selective PI3Kα inhibitor pictilisib (GDC-0941) or the selective AKT1/2 inhibitor ipatasertib (GDC-0068) after 96-h treatment (range 0,05–10 μmol/L) evaluated for proliferation by MTT assay, as described in the Materials and Methods. All the results are average ± SD of 3 independent experiments, each done in triplicate. *: p < 0.05. **c**, combination of cetuximab and refametinib (BAY-86-9766), used at the fixed ratio 1 μg/ml:1 μmol/L, with pictilisib (GDC-0941), used at the IC_50_ ratio, as described in the Chou-Talalay model of synergism. Values on the X-axis refer to cetuximab and refametinib. Cetuximab:Refametinib:Pictilisib ratios are, respectively: 16 μg/ml:16 μmol/L:1 μmol/L for HCT116 CM-R, 150 μg/ml:150 μmol/L:1 μmol/L for HCT15 CM-R, 2 μg/ml:2 μmol/L:1 μmol/L for LOVO CM-R, 2 μg/ml:2 μmol/L:1 μmol/L for SW480 CM-R. **d,** Combination index (C.I.) analysis of the combination between cetuximab, refametinib and pictilisib in the resistant cell lines at different Effective Doses (EDs). CI < 1 indicates synergism, while CI < 0.5 indicates strong synergism

In order to assess the mechanisms responsible for the onset of resistance to the vertical suppression of the EGFR/MAPK pathway, intracellular signaling molecules were studied using immunoblot assay. The activation of PI3K/AKT pathway and their downstream effectors S6RP and 4eBP1 is a consistent event across the resistant clones (Fig. 2a). To this purpose, selective PI3Kα and AKT1/2 inhibition were obtained with, respectively, pictilisib (GDC-0941) and ipatasertib (GDC-0068). With the exception of HCT15, whose known PIK3CA E545K mutation predicts a better response to AKT-inhibitors, no significant difference in sensitivity to PI3K/AKT inhibitors were observed between resistant and parental cell lines (Fig. 2b). Nevertheless, PI3K/AKT pathway blockade using pictilisib is able to revert resistance to EGFR and MEK combined inhibition, inducing a shift in cellular mortality, when used in combination with cetuximab and refametinib according to IC_50_ ratio, as described in the Chou-Talalay model [24] (Fig. 2c). Combination index analysis indicates that pictilisib is synergistic when used with cetuximab and refametinib, as shown in Fig. 2d.

### Role of receptor tyrosine kinases (RTK) upregulation and transcriptional adaptation in resistant cell lines

**Fig. 3:**
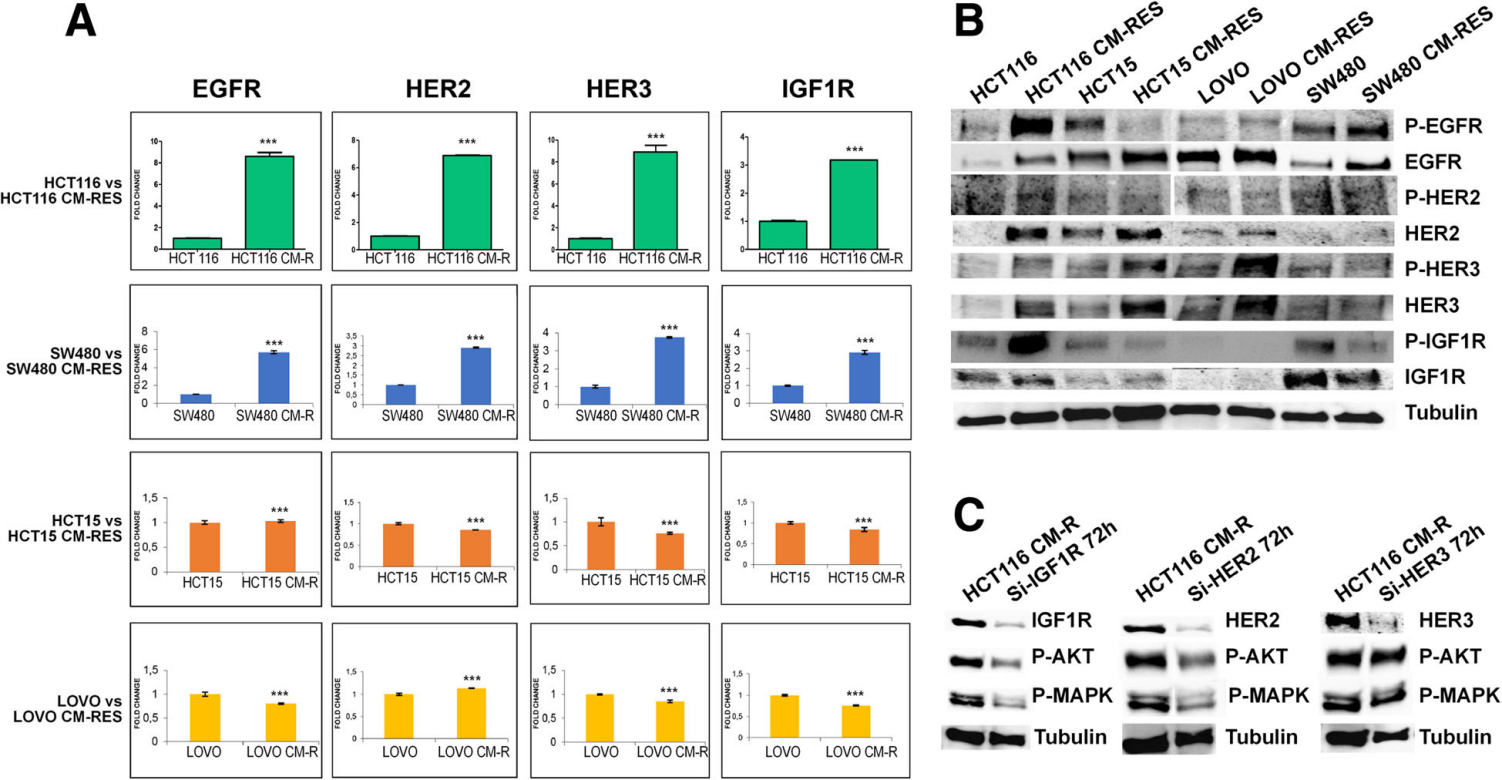
Receptor tyrosine kinase (RTK) expression. **a**, quantitative RT-PCR on transmembrane receptors in HCT116, SW480 and HCT15 resistant cells compared with parental cell clones. Bars indicate fold-change compared to parental control. ***: p < 0.001. **b**, western blot analysis of RTKs and their phosphorylation status at baseline conditions in parental versus resistant cell lines. Tubulin was used for normalization of protein extract content. **c**, western blot analysis at 72 h after silencing of IGF1R, HER2 or HER3, showing the level of phosphorylation of the downstream effectors AKT and MAPK. Tubulin was used for normalization of protein extract content

To investigate the putative mechanism of activation of PI3K axis in our model, receptor tyrosine kinases expression and activation status were studied using quantitative real-time PCR and immunoblot, respectively (Fig. 3a-b). Upregulation of HER family receptors and IGF1R is a common event in resistant clones, albeit with several differences. In particular, EGFR, HER2, HER3 and IGF1R result transcriptionally upregulated in HCT116 and SW480 resistant clones compared to parental cell lines, while no major differences are evident in HCT15 resistant cell lines (Fig. 3a). Western blot analysis showed an increased phosphorylation of EGFR, HER2, HER3 and IGF1R, which is particularly evident in HCT116 CM-R (Fig. 3b). Moreover, we analyzed baseline microarray gene expression of parental HCT116 and LOVO cell lines as compared to resistant clones, with the aim to identify genes or pathway related with the resistant phenotype. In this respect, we found 1755 and 658 genes upregulated and downregulated respectively in HCT116 CM-R cell line and 1177 and 625 genes upregulated and downregulated in LOVO CM-R cell line (t-test, *p* < 0,05) (data not shown). Among the upregulated genes, there was a consistent genetic dysregulation in genes involved in EGFR/ErbB, insulin and MAPK signaling pathways (Table 2).

**Table 2:** Significant upregulated genes in resistant cell lines

HCT116 CM-R vs HCT116			LOVO CM-R vs LOVO		
Gene Name	Gene Symbol	Fold change	Gene Name	Gene Symbol	Fold change
Neurofibromin 1	*NF1*	7,860,121	Insulin-like growth factor binding protein 7	*IGFBP7*	786,601
Erb-b2 receptor tyrosine kinase 3	*ERBB3*	6,631,199	Insulin receptor substrate 2	*IRS2*	7,395,454
Epidermal growth factor receptor	*EGFR*	5,149,977	Amphiregulin	*AREG*	4,963,224
E-cadherin	*CDH1*	3,811,482	Epidermal growth factor receptor	*EGFR*	455,673
Erbb2 interacting protein	*ERBB2IP*	3,587,709	Epiregulin	*EREG*	4,104,191
Insulin induced gene 2	*INSIG2*	3,514,022	Insulin induced gene 1	*INSIG1*	3,779,914
Insulin induced gene 1	*INSIG1*	2,569,083	Insulin induced gene 2	*INSIG2*	3,444,767
Erb-b2 receptor tyrosine kinase 2	*ERBB2*	2,387,238	Vimentin	*VIM*	290,101
Mitogen-activated protein kinase kinase kinase 10	*MAP3K10*	2,319,185	E-cadherin	*CDH1*	2,866,793
V-akt murine thymoma viral oncogene homolog 2	*AKT2*	2,270,916	V-akt murine thymoma viral oncogene homolog 3	*AKT3*	2,849,459
Transforming growth factor alpha	*TGFA*	2,015,933	Transforming growth factor alpha	*TGFA*	2,341,766
Insulin receptor substrate 2	*IRS2*	2,009,206	ERBB receptor feedback inhibitor 1	*ERRFI1*	2,141,185

Microarray analysis on HCT116 and LOVO resistant vs parental cell lines show recurrent upregulations of genes involved in HER, Insulin and AKT signaling

Finally, transient knock-down of HER2 and IGF1R, commonly associated to PI3K/AKT pathway activation [25], showed to decrease the activation levels of both AKT and MAPK in the resistant cell lines, while HER3 knockdown is not sufficient to suppress the activation of AKT and MAPK (Fig. 3c).

### Mechanisms of activation of RTK in resistant cell lines

**Fig. 4:**
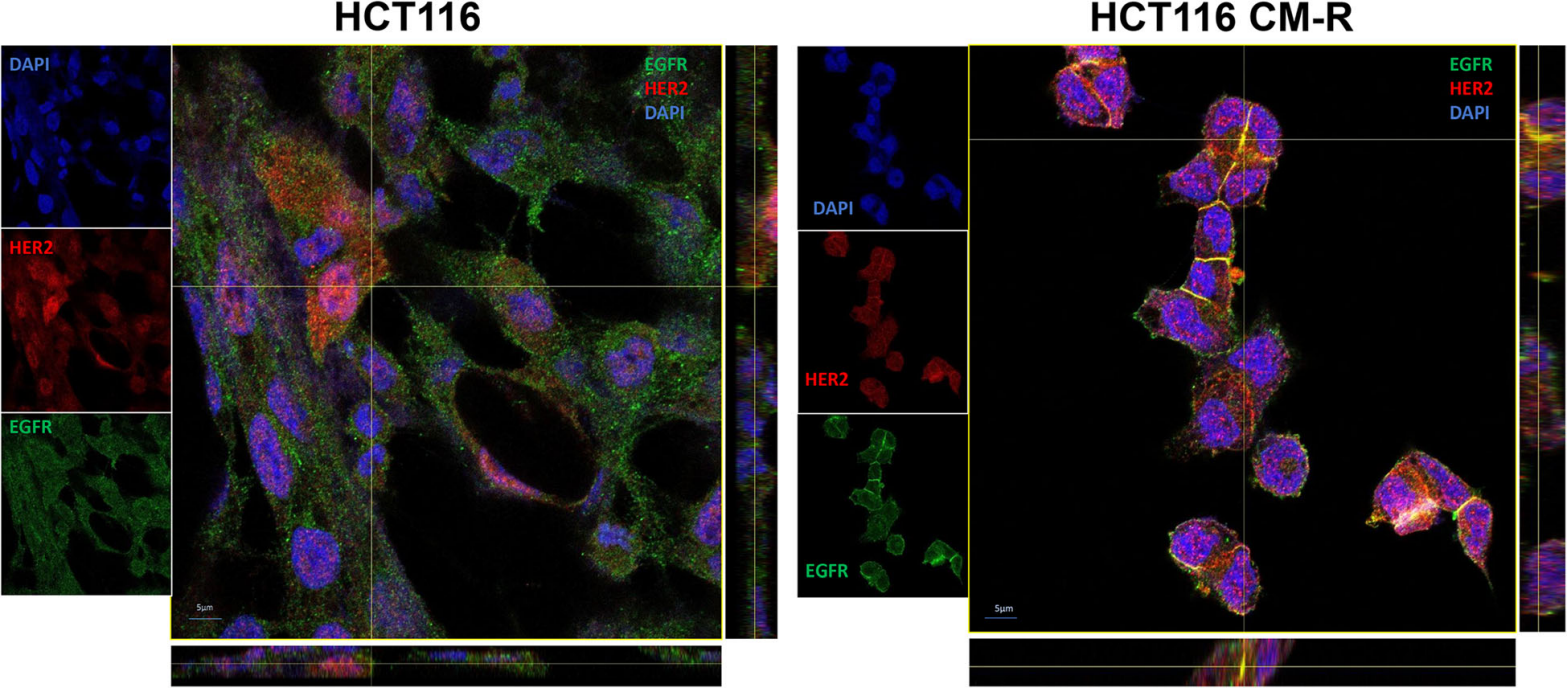
Heterodimerization of RTK. Indirect immunofluorescence for EGFR (green) and HER2 (red) and their merge with DAPI in HCT116 vs HCT116 CM-R; z-stacking analysis reveals co-localization (yellow dots) of the two receptors on cell membranes at multiple focal planes electively in HCT116 CM-R cells

With the aim to figure out the mechanism of activation of RTKs in resistant cell lines compared to parental cell lines, the role of ligands and receptor interaction was explored. To this respect, microarray analysis showed that, beside the upregulation of the receptors, ligands upregulation is also evident in our model. In particular, TGFα transcription is doubled in both cell lines analyzed, while epiregulin and amphiregulin result upregulated by 4 and almost 5-fold, respectively, in LOVO CM-R and HCT116 CM-R (Table 2). Moreover, we performed indirect immunofluorescence, in order to analyze the distribution of RTKs in our resistant cell lines. In particular, in HCT116 CM-R line, the distribution of EGFR and HER2 is prevalent on the cell membrane, where their co-localization is evident at multiple focal planes, as confirmed by z-stacking of the images (Fig. 4).

## Discussion

**Fig. 5:**
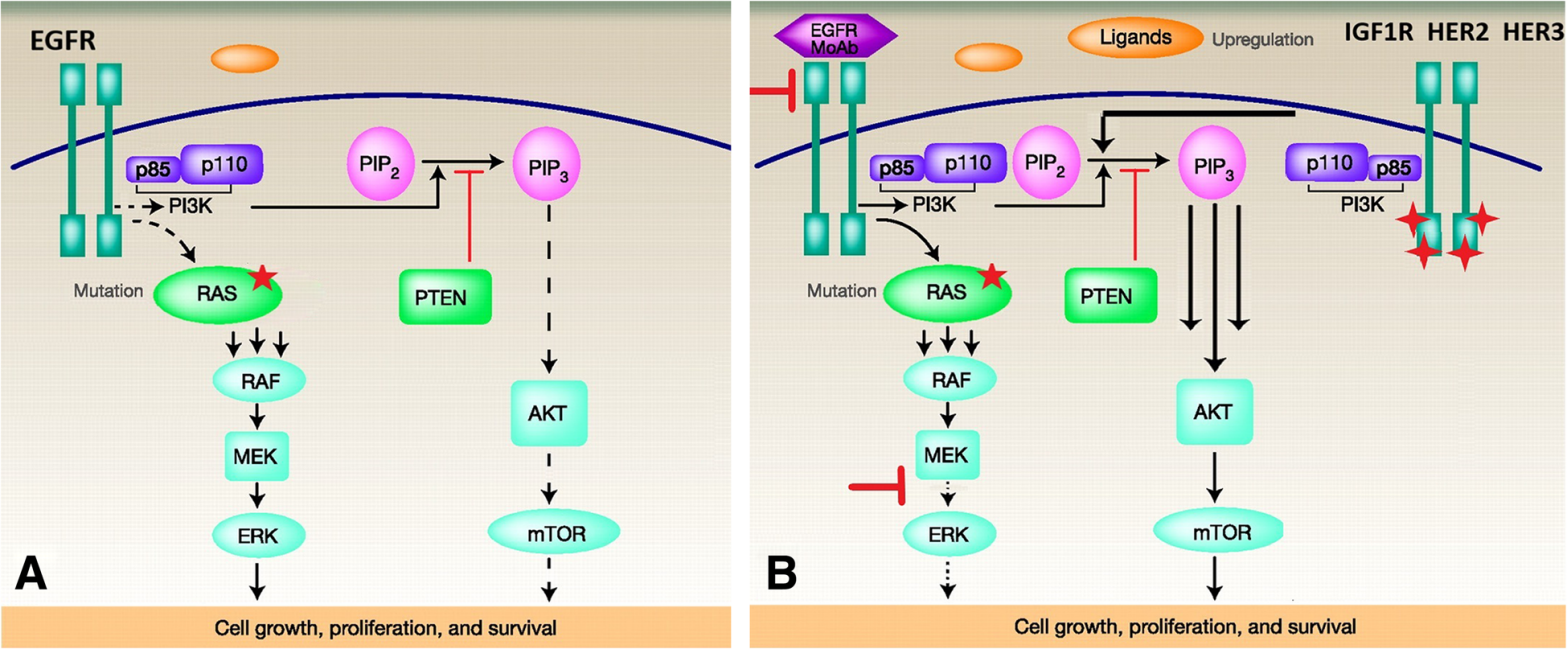
Proposed model of PI3K activation in CM-RES clones. **a**, in baseline conditions, the presence of activating KRAS mutations causes a sustained MAPK signaling independently from EGFR stimulation. **b**, after prolonged inhibition of the MAPK pathway through vertical suppression of EGFR and MEK, the compensatory upregulation of RTKs and their ligands generates a preferential association of RTK with PI3K

Understanding the molecular mechanisms that lead to resistance to anticancer therapies is critical in order to develop more effective therapies. KRAS mutations play a prominent role in the landscape of anti-EGFR therapy primary and acquired resistance and still constitute an unmet therapeutic need, since no specific treatment is currently available to the relevant subgroup of mCRC patients that carry these mutations [3]. After the initial enthusiasm brought by the availability of several MEK inhibitors, it was promptly shown that MEK inhibition alone is not capable to suppress MAPK pathway in RAS-mutated cancers and that co-targeting of upstream receptors might be a way to avoid pathway reactivation [26]. Two seminal studies published in 2014 [21, 22] have shown that vertical suppression by combined blockade of EGFR and MEK is able to overcome primary and acquired resistance to anti-EGFR agents, including the case of KRAS mutations. Both studies conclude that, although significant and sustained, the response to vertical suppression of the EGFR/MAPK pathway is only transient and thus limited by the onset of acquired resistance. In agreement with these findings, in the present work we have generated 4 different human KRAS-mutated CRC cell lines resistant to the combination of the anti-EGFR cetuximab and the MEK-inhibitor refametinib. These resistant clones exhibit different morphological characteristics compared to parental cell lines, but do not show new genetic alterations underlying resistance onset. Activation of AKT and its downstream effectors is a consistent feature across the four different cell lines considered in this work, though pharmacologic inhibition of either PI3Kα or AKT1/2 using pictilisib and ipatasertib, respectively, is able to revert the resistance phenotype in our model. Nevertheless, PI3K/AKT axis constitutes the principal survival pathway in the resistant cell lines, as combination of pictilisib with cetuximab and refametinib restores the sensitivity to the vertical suppression to the levels of parental cell lines, irrespectively of the PIK3CA mutational status, and is significantly synergistic as evidenced by the combination index analysis. This phenomenon is probably underpinned by a wide transcriptional rearrangement in these cell clones that allows for a rapid switch towards the PI3K/AKT pathway only when EGFR/MAPK pathway is fully suppressed, as a consequence of prolonged MAPK suppression and c-myc persistent deregulation, as suggested in another publication on breast cancer [27]. Previous studies have showed how, in KRAS-mutant CRC, PI3K activation is mediated by RTK signaling, despite KRAS being capable of cross-activating PI3K [20, 28, 29], for this reason we investigated the status of multiple receptors. Among the upregulated and activated receptors in our system, EGFR, HER2, HER3 and IGF1R are the ones able to activate effectively PI3K signaling. In fact, both HER2 and IGF1R knockdown is associated to decreased AKT and MAPK phosphorylation, demonstrating that they are actually responsible for both PI3K/AKT and MAPK pathway activation. On the other hand, even if HER3 upregulation and activation are clearly evidenced, its knockdown is not able to suppress the activation of PI3K/AKT and MAPK pathways; in this context it can be postulated that in the absence of HER3, HER2 heterodimerization with EGFR, as clearly shown in our system (Fig. 4), is able to compensate the activation of the downstream PI3K and MAPK pathways. Moreover, microarray data evidence how not only RTKs but also ligands in the HER family pathway are upregulated in the resistant cell lines analyzed, suggesting the existence of feedback mechanisms that lead to autocrine loops and sustain the activation of different RTKs, as previously evidenced by other authors in different models [30, 31]. The transcriptional rearrangement that is ultimately responsible for RTK-PI3K axis activation also involves cellular shape and motility. In our model, cell lines that become resistant to the combination of anti-EGFR and MEK-inhibitor display increased epithelial features when compared to parental cell lines. Although resistance to therapy is commonly associated to the more aggressive mesenchymal phenotype, it was previously demonstrated that the unbalance between RAS/MAPK and PI3K/AKT signaling in favor of the latter decreases the EMT potential in cancer cells, even in presence of EMT inductors such as Snail [32]. This might be the cause of the discrepancy between resistance to therapy and a more prominent epithelial phenotype in resistant clones. In fact, on the functional level, resistant clones show a decreased migration and invasion potential. Finally, the model depicted in Fig. 5 illustrates how different mechanisms cooperatively converge on PI3K/AKT pathway activation when EGFR/MAPK pathway is inhibited: upregulation and activation of different receptors of the HER family and IGF1R, increased heterodimerization of these receptors, upregulation of HER and IGF family ligands.

## Conclusions

The data presented in this work increase our understanding of the molecular mechanisms that lead to acquired resistance to targeted therapies in CRC. We have shown how transcriptional adaptation is ultimately responsible for PI3K activation upon vertical suppression of the EGFR/MAPK pathway through the upregulation and cooperative activation of different RTKs in a preclinical model of KRAS-mutated colorectal cancer. This data, taken together, explain why vertical suppression of EGFR/MAPK pathway has only a transient effect in KRAS-driven colorectal cancers and stimulate new research on the best treatment approach for this aggressive cancer type, encouraging further evaluation of novel combination strategies including PI3K inhibitors.

## Abbreviations

ATTC: American Type Culture Collection
CM-R: Cetuximab-MEK-inhibitor-resistant
CRC: Colorectal cancer
DMSO: Dimethylsulfoxide
ED50 / 75 / 90: Effective Dose 50 / 75 / 90
EGFR: Anti-epidermal growth factor receptor
FITC: Fluorescein isothiocyanate
IC_50_: Half-maximal inhibitory concentration
moAb: Monoclonal antibody
MTT: 3-(4, 5-dimethylthiazol-2-yl)-5-(3-carboxymethxyphenyl)-2-(4sulfophenyl)-2H-tetrazolium
qRT-PCR: Quantitative Reverse Transcriptase Polymerase Chain Reaction
RTK: Receptor Tyrosine Kinase
siRNA: Small inhibitor RNAs
WB: Western blot
WT: Wild type
